# A case-referent study: light at night and breast cancer risk in Georgia

**DOI:** 10.1186/1476-072X-12-23

**Published:** 2013-04-17

**Authors:** Sarah E Bauer, Sara E Wagner, Jim Burch, Rana Bayakly, John E Vena

**Affiliations:** 1 Department of Epidemiology and Biostatistics, College of Public Health, University of Georgia, Athens, GA, Georgia; 2 Department of Epidemiology and Biostatistics, Cancer Prevention and Control Program, University of South Carolina, Columbia, SC, USA; 3 Georgia Department of Public Health, Georgia Comprehensive Cancer Registry, Atlanta, GA, Georgia

**Keywords:** Light at night (LAN), Artificial LAN, Breast cancer, Circadian disruption

## Abstract

**Background:**

Literature has identified detrimental health effects from the indiscriminate use of artificial nighttime light. We examined the co-distribution of light at night (LAN) and breast cancer (BC) incidence in Georgia, with the goal to contribute to the accumulating evidence that exposure to LAN increases risk of BC.

**Methods:**

Using Georgia Comprehensive Cancer Registry data (2000–2007), we conducted a case-referent study among 34,053 BC cases and 14,458 lung cancer referents. Individuals with lung cancer were used as referents to control for other cancer risk factors that may be associated with elevated LAN, such as air pollution, and since this cancer type was not previously associated with LAN or circadian rhythm disruption. DMSP-OLS Nighttime Light Time Series satellite images (1992–2007) were used to estimate LAN levels; low (0–20 watts per sterradian cm^2^), medium (21–41 watts per sterradian cm^2^), high (>41 watts per sterradian cm^2^). LAN levels were extracted for each year of exposure prior to case/referent diagnosis in ArcGIS.

**Results:**

Odds ratios (OR) and 95% confidence intervals (CI) were estimated using logistic regression models controlling for individual-level year of diagnosis, race, age at diagnosis, tumor grade, stage; and population-level determinants including metropolitan statistical area (MSA) status, births per 1,000 women aged 15–50, percentage of female smokers, MSA population mobility, and percentage of population over 16 in the labor force. We found that overall BC incidence was associated with high LAN exposure (OR = 1.12, 95% CI [1.04, 1.20]). When stratified by race, LAN exposure was associated with increased BC risk among whites (OR = 1.13, 95% CI [1.05, 1.22]), but not among blacks (OR = 1.02, 95% CI [0.82, 1.28]).

**Conclusions:**

Our results suggest positive associations between LAN and BC incidence, especially among whites. The consistency of our findings with previous studies suggests that there could be fundamental biological links between exposure to artificial LAN and increased BC incidence, although additional research using exposure metrics at the individual level is required to confirm or refute these findings.

## Background

Increasing urban development and the subsequent need for artificial lighting of roadways, shopping centers, and homes, has diminished the daily dark period [1]. Artificial light sources have the power to light the evening sky up to 200 thousand times brighter than the natural new moon [2]. The *First Atlas of Artificial Night Sky Brightness*, reports that 99% of American and European populations, and nearly one-fifth of world terrain, is under light-polluted skies [3]. Rising concern has begun to mount about the detrimental health effects of our newly created electrified environment.

Although the origins of elevated global breast cancer (BC) rates are not fully understood, the highest incidence is observed in industrialized nations [4]. The hypothesis that light at night (LAN) and corresponding decreases in nocturnal melatonin production may act as a BC risk factor [5] has received increasing attention over the past decade. This hypothesis proposes that exposure to LAN disrupts endogenous melatonin production. Melatonin (MLT) is considered an oncostatic or anti-estrogenic agent that is suppressed by ambient light exposure via the retinohypohalamic pineal tract. MLT may affect estrogen activity via several processes and elevated estrogen may be a risk factor for BC [6]. Therefore, reductions in MLT resulting from LAN exposure may promote BC development [7-9] due to the facilitation of increased estrogen production [10-16]. Several prospective studies have reported increased BC risks among women with elevated circulating estrogen concentrations or reduced MLT levels. Shift work, which is associated with both LAN exposure and reductions in MLT production, has been linked with BC and has been designated by the International Agency for Research on Cancer (IARC) as a Group 2A probable human carcinogen [17].

The purpose of this case-referent study was to test the hypothesis that an increased incidence of BC is associated with residence in highly illuminated areas, as defined by time series satellite imagery, in Georgia (GA). We hypothesized that higher LAN levels would be associated with BC incidence, and not, or to a lesser extent, with lung cancer. An elevated incidence of lung cancer related to LAN is not expected because lung cancer is not estrogen dependent and thus, was included in the present analysis as referent cases [14]. Cases and referents were analyzed in relation to residential location and corresponding average LAN exposure for years prior to cancer diagnosis, dating back to 1992, the earliest year of exposure data. Infrared light detected by a nighttime satellite was used to assess exposure, assuming that the more brightly lit areas contained residents who, on average, have had greater exposure to circadian-disruptive light than residents in the lesser lit areas. Additionally, the variation between geographic trends of LAN and BC incidence were evaluated statewide and over time. We also examined whether the relationship between elevated LAN exposure and BC incidence was modified by race. To the best of our knowledge, this is one of the first studies to explore LAN and BC risk among racial subgroups. Previous studies suggest that African Americans may be more susceptible to circadian misalignment. African Americans have a shorter circadian period, larger phase advances, and smaller phase delays relative to Caucasian subjects [18].

## Results

In total, 47,817 incident cancers, 33,503 breast and 14,314 lung were identified for inclusion in the study. Analyses were restricted to cancer of the female breast and lung, thus, 302 male BC cases and 26,296 male lung cancer cases were excluded. Breast and lung cancer cases lacking address-level geocoding accuracy were excluded from analysis (N = 12,618). The frequencies of individual, county, and public health district variables were compared by case status using Chi-square tests. As expected, lung cancers were diagnosed at more advanced stages and grades of tumor development. More BC cases were diagnosed with lower grade (grade 1 and 2) tumors (18% and 35%, respectively; Table 1), while the majority of referents presented grade 3 or ‘other’ tumor classification upon diagnosis (29% and 54%, respectively; Table 1). Cases were most frequently diagnosed with local stage tumors (60%), whereas 45% of referents were diagnosed with distantly staged tumors. Cases had a higher mean LAN exposure of 35 watts per cm^2^ per sterradian, opposed to 18 watts per cm^2^ per sterradian for referents (Table 1). There were no other notable differences among cases and referents.

**Table 1:** Descriptive characteristics of breast cancer and lung cancer cases and referents diagnosed in years 2000–2007 (N = 61,129)

***Individual-level variables***	**Cases (N = 42,754)**		**Referents (N = 18,375)**	
	**N**	***%***	**N**	***%***
**LAN mean value (watts per cm**^**2 **^per sterradian)*				
High (> 41)	27,121	*71*	10,970	*60*
Medium (21–41)	5,974	*14*	2,623	*14*
Low (0–20)	9,659	*23*	4,782	*26*
Race*				
White	31,638	*75*	14,885	*82*
Black	10,461	*25*	3,364	*18*
Geocoding Match Status*				
Matched	34,053	*80*	14,458	*79*
Unmatched	8,701	*20*	3,917	*21*
Tumor Grade*				
Grade 1	7,496	*18*	785	*4*
Grade 2	15,117	*35*	2,411	*13*
Grade 3	14,741	*34*	5,207	*29*
Other	5,348	*13*	9,915	*54*
Tumor Stage*				
Distant	1,992	*5*	8,230	*45*
Local	25,732	*60*	3,665	*20*
Regional	13,690	*32*	4,697	*25*
Unstaged	1,340	*3*	1,783	*10*
	**Mean (SD)**		**Mean (SD)**	
**Mean age at diagnosis**	60 (14)		68 (12)	
**Mean LAN value (watts per cm**^**2 **^**per sterradian)**	35 (292)		18 (491)	
*County-level variables*	**Cases (N = 34,053)**^**†**^		**Referents (N = 14,458)**^**†**^	
Average family size*				
≥ 3.17	25,206	*74*	10,103	*70*
< 3.17	8,847	*26*	4,328	*30*
Median household Income* (quartiles)				
< $27,869	982	*3*	486	*3*
≥ $27,869 - $31,950	3,227	*10*	1,546	*11*
> $31,950 - $38,799	8,296	*24*	4,098	*28*
> $38,799	21,548	*63*	8,328	*58*
MSA Status* (2003)				
MSA	28,523	*84*	11,757	*81*
Non-MSA	5,530	*16*	2,701	*19*
Annual PM 2.5 level* (quartiles)				
< 11.4	2,076	*6*	1,016	*7*
≥ 11.4-13.1	3,202	*9*	1,514	*10*
>13.1-15.2	7,016	*21*	3,282	*23*
>15.2	21,759	*64*	8,646	*60*
Per 1,000 women aged 15–50, number of births in last year* (quartiles)				
< 40	2,621	*8*	1,211	*8*
≥ 40 - 54	7,217	*21*	3,299	*23*
> 54 – 66	18,384	*54*	7,066	*49*
> 66	5,831	*17*	2,882	*20*
Total population living in MSA/PMSA, percentage living in different residence in 1995*				
No change in residence since 1995	8,693	*26*	4,372	*30*
Change in residence since 1995	25,360	*74*	10,086	*70*
Percent female smokers, public health district level* (quartiles)				
< 19.5%	13,113	*39*	4,783	*33*
≥ 19.5% - 21.4%	9,907	*29*	4,165	*29*
> 21.4% - 22.3%	2,814	*8*	1,271	*9*
> 22.3%	8,219	*24*	4,239	*29*
	**Mean (SD)**		**Mean (SD)**	
**Percentage of population below the poverty line**	14.43 (5.64)		14.79 (5.57)	
**Percentage of African-American females**	29.60 (17.92)		28.29 (17.92)	
**Percent high school graduate or higher**	84.00 (6.11)		83.05 (6.38)	
**Percentage of population over 16 in labor force**	66.58 (5.64)		65.79 (5.72)	
**Percentage of population in different residence in 1995**	48.07 (5.29)		47.35 (5.46)	

Abbreviations: *LAN*, Light at night in watts per cm^2^ per sterradian; *SD*, Standard Deviation; *MSA*, Metropolitan Statistical Area; *PM*, Particulate Matter_2.5_.

Case: (breast cancer); Referent (lung cancer).

Match Status: Only cancer cases registering address level accuracy (AI0-AX3) were included for analysis.

Annual PM 2.5 level; annual average ambient concentrations of PM 2.5 in micrograms per cubic meter, based on seasonal averages and daily measurement, 2001.

* Statistically significant chi-square result (p < 0.05).

^**†**^ Number of cases and referents differ from individual-level analysis figures; some responses regarding county location were missing and dropped from analysis.

**Figure 1:**
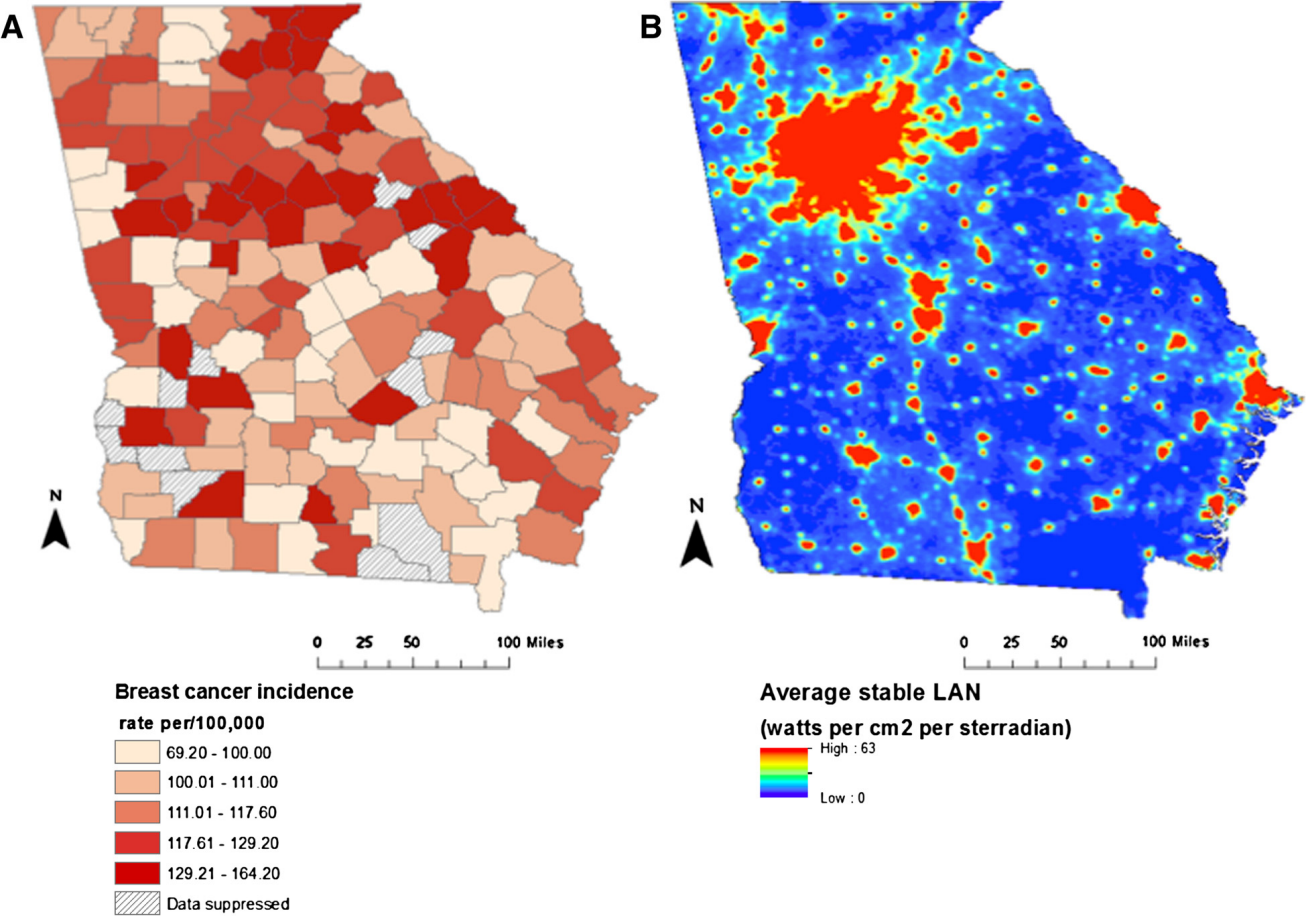
**Breast cancer incidence by county and Light at Night Exposure for Georgia. A.** Spatial patterns: breast cancer incidence by county, all races, 2005–2009. **B.** Light at Night Exposure for Georgia, 2007.

A comparative evaluation of spatial variation between county-level BC incidence and LAN exposure revealed strikingly similar geographic trends across the state of GA (Figure 1). Regionally, elevated BC incidence rates were observed in northwestern and north central GA. Elevated incidence rates were also observed along the southeast border and in more isolated pockets in the southwest corner of the state. Similar to the county-level BC incidence map, elevated LAN was also documented in the north central region, as well as in isolated pockets along the southeast border and southwest corner of the state. Areas of elevated LAN exposure and BC incidence correspond to urban centers of which, the Atlanta metropolitan area was the most noteworthy.

**Figure 2:**
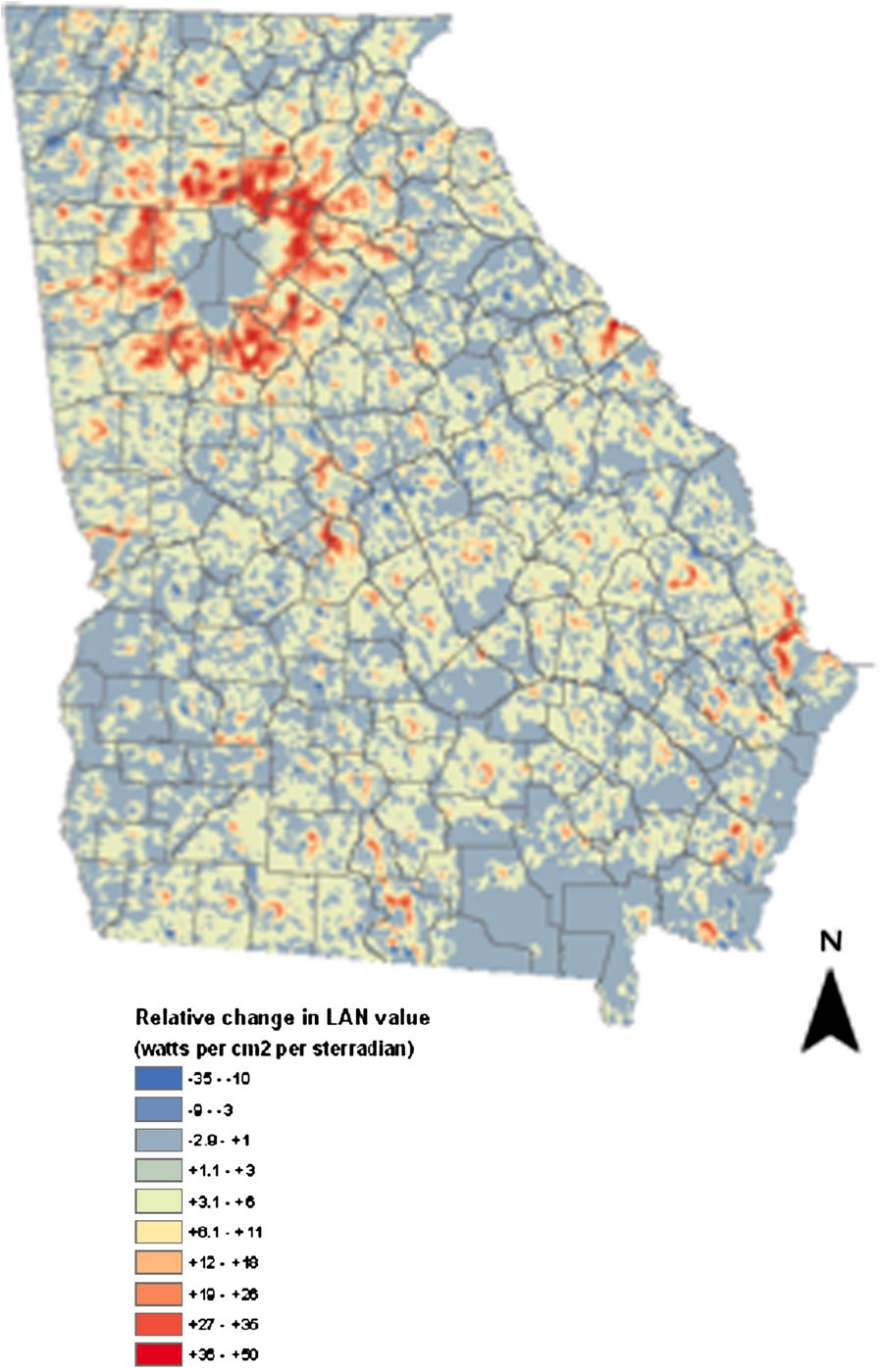
Relative change in light at night values, 2007 versus 1992.

Geographic trends in LAN exposure between the most recent (2007) and the oldest (1992) years of data were also evaluated (Figure 2). The most noteworthy area of increasing LAN occurred in north central GA, in areas surrounding central Atlanta. Red shaded regions indicate areas of increasing LAN over time. The most prominent areas of increasing LAN surround central Atlanta and represent increasing urbanization. Figure 2 obviates the sprawl of the Atlanta metropolitan area, and the successive increases in light pollution.

Adjusted odds ratios for BC indicated that cases were 1.12 times more likely to have been diagnosed if exposed to elevated LAN levels, as opposed to low LAN levels (OR = 1.12, 95% CI [1.04, 1.20]; Table 2). When the analyses were stratified by race, LAN was not associated with BC among blacks for, high vs. low (OR =1.02, 95% CI [0.82, 1.28]) or medium vs. low LAN levels (OR = 1.04, 95% CI [0.78, 1.38]; Table 3). A positive association between LAN levels and BC case status was observed among whites; women with BC were 13% more likely to live in areas with high LAN compared to those with low LAN levels (OR = 1.13, 95% CI [1.05-1.22]; Table 3).

**Table 2:** Unadjusted and adjusted results for the association between light at night and breast cancer risk

	N (%)*		**Crude results (n = 61,129)**		Adjusted results** (n = 47,817)	
***LAN exposure***	**Cases**	**Referents**	**Odds ratios**	**95% CI**	**Odds ratios**	**95% CI**
Low	27,121 (63)	10,970 (60)	*Ref.*		*Ref.*	
Medium	5,974 (14)	2,623 (14)	1.13	(1.07-1.19)	1.06	(0.97-1.16)
High	9,659 (23)	4,782 (26)	1.22	(1.18-1.28)	1.12	(1.04-1.20)^†^

*Values reflect sample prior to geocoding match and county-level variable exclusions.

**Model adjusted for: race, tumor grade and stage, year of diagnosis, age at cancer diagnosis, Metropolitan Statistical Area (MSA) status, births per 1,000 women aged 15–50, MSA population mobility, population over 16 in the labor force, and prevalence of cigarette smoking.

Case (breast cancer); Referent (lung cancer).

† Statistically significant with p < 0.05.

Abbreviations: *LAN*, Light at night in watts per cm^2^ per sterradian; *OR*, odds ratio; *CI*, confidence interval; *MSA*, metropolitan statistical area.

Matched data displayed; only cancer cases registering address level accuracy (AI0-AX3) were included for analysis.

**Table 3:** Adjusted results for the association between light at night and breast cancer risk with racial stratification*

	N (%)*		**Crude results (n = 60,348)**		Adjusted results** (n = 47,817)	
***LAN exposure***	**Cases**	**Referents**	**Odds ratios**	**95% CI**	**Odds ratios**	**95% CI**
***Whites***						
Low	8,367 (26)	4,383 (29)	*Ref.*		*Ref.*	
Medium	4,912 (16)	2,320 (16)	1.11	(1.04, 1.18)	1.07	(0.97-1.17)
High	18,359 (58)	8,182 (55)	1.18	(1.12, 1.23)	1.13	(1.05 -1.22)^†^
***Blacks***						
Low	1,240 (12)	387 (11)	*Ref.*		*Ref.*	
Medium	991 (9)	292 (9)	1.06	(0.89, 1.26)	1.04	(0.78-1.38)
High	8,230 (79)	2,685 (80)	0.96	(0.85, 1.08)	1.02	(0.82-1.28)

*Values reflect sample prior to geocoding match and county-level variable exclusions.

**Model adjusted for: tumor grade and stage, year of diagnosis, age at cancer diagnosis.

Metropolitan Statistical Area (MSA) status, births per 1,000 women aged 15–50, MSA.

population mobility, population over 16 in the labor force, and prevalence of cigarette smoking.

Case (breast cancer); Referent (lung cancer).

† Statistically significant.

Abbreviations: *LAN*, Light at night in watts per cm^2^ per sterradian; *OR*, odds ratio; *CI*, confidence interval;

*MSA*, metropolitan statistical area.

Matched data displayed; only cancer cases registering address level accuracy (AI0-AX3).

were included for analysis.

## Discussion

The present analyses were based on a case-referent study of 48,511 female cancer cases (34,053 breast and 14,588 lung) and LAN levels averaged prior to cancer diagnosis. Study findings suggest that elevated LAN was associated with an increased odds of BC risk among women after controlling for year of diagnosis, race, tumor grade and stage, age at diagnosis, MSA status, births per 1,000 women aged 15–50, MSA population mobility, female smoking rate, and population over 16 in the labor force. The results are consistent with a previous study that observed a spatial trend of elevated BC in urban areas, which includes the majority of GA’s residential population (71%) [19].

It has been hypothesized that disruption of melatonin by LAN can promote BC development [5]. Melatonin suppression [20,21] may promote tumor growth [22] via several possible mechanisms including an increase in estrogen secretion. Although the exact mechanism remains to be described, light exposure may act on tumor formation and growth via a direct oncostatic action, through interference with estrogen receptor function, by affecting thermoregulatory and immune function, and by altering free radical biology (reviewed in [23]). It is also possible that light alters circadian rhythm generation in the suprachiasmatic nuclei (SCN), which has the potential for disruption of clock gene communication with cell cycle regulation in the mammary tissue [24,25].

This study is the first to examine the effects of LAN across racial subgroups. Contrary to our hypothesis, a relationship between LAN and BC incidence was only observed among white women. These results were unexpected given the experimental evidence suggesting that blacks may be more susceptible to circadian misalignment, due to a shorter circadian period, larger phase advances, and smaller phase delays relative to white subjects [18]. The possible set of mechanisms responsible for the observed racial disparity is likely complex, and to date, poorly understood.

Although the level of melatonin production among blacks relative to whites is unclear, racial differences in naturally occurring melatonin production and secretion have been proposed [26]. Sensitivity of melatonin to light suppression may be influenced by eye pigmentation, which can vary due to race or ethnicity [27]. The percentage of melatonin suppression secretion after light exposure was significantly larger in light-eyed compared to individuals with darker eye pigmentation [27]. The common occurrence of lighter iris color is found almost exclusively among Caucasians [28]. Based on these observations, white women may be at increased risk of melatonin suppression from exposure to LAN, although additional research is needed to elucidate the possible consequences of racial differences in the biological processing of LAN.

Our study findings are especially important given GA’s increasing trajectory of urban development. GA was the fourth fastest-growing state between 2000 and 2010 with an increase of 1.5 million residents. Much of that growth was centrally focused in Atlanta and its surrounding metropolitan area, which accounted for over two-thirds (68%) of the state’s population growth during the last decade (Figure 2) [29]. The expansion of the Atlanta metropolitan region will only increase LAN, and could exacerbate the already increased BC rates observed in this growing urban region. Figure 2 confirms Atlanta to be the most noteworthy area of increasing LAN, shown by the circular red area surrounding the core of Atlanta. The core of Atlanta is blue, which translates to no LAN increase. Yet, this is likely due to measurement error because the core urban area registered the maximum LAN value measurable by DMSP-OLS satellites in 1992 (63 watts per cm^2^ per sterradian), therefore it does not appear that there has been an increase in light. However, this area has continued to expand, subsequently increasing LAN levels.

When using a satellite image to proxy the light intensity on the ground level, there is understandable concern about the accuracy by which the spatial area depicted on the images represents the size of the lit area on the ground. Imagery from the DMSP-OLS satellite has a tendency to overestimate ground illumination due to coarse spatial resolution, large overlap between pixels, errors in geolocation, or atmospheric water vapor content [30]. Pixel misclassification is exacerbated in the stable light images by counting all occasions of a lighted pixel and by registration errors around persistently lit regions [31]. The combined effect of these factors ultimately results in a general overestimation of the illumination in the area of study. This can be especially problematic when assigning exposure variables to cases and referents, and may result in non-differential misclassification. However, non-differential exposure misclassification, if present, would attenuate rather than exaggerate our results.

The DMSP output from the satellite may not strictly correlate with the restricted portion of the spectrum that is circadian disruptive. Novel ocular studies have identified that MLT suppression is wavelength dependent [32-37] and have defined the visible short-wavelength sensitivity of the human melatonin suppression action spectrum [33,37-39]. We conducted an ancillary light validation study to quantify the relationship between presence of circadian-effective ambient outdoor light levels and our exposure variable. This light validation study was conducted in Athens, Georgia using the Daysimeter. The Daysimeter is a device that records ambient light levels that stimulate both the visual and circadian systems [40]. It has previously been used to characterize circadian specific wavelengths, or circadian light [41], and may also help estimate the magnitude of effect on melatonin suppression. This study aimed to describe the relationship between circadian light (CL) at ground level and satellite photometry in the local area (unpublished observations from: Perry G, Bauer S, Wagner S, Vena J). The findings suggested that ground level CL and satellite photometry are significantly correlated (p-value = 0.0003). Our primary interest was to isolate the role the external environment may play on BC risk, although a closer look at the indoor sleeping habitat or nocturnal behavior patterns may prove to be more influential on BC risk than the external light environment. Yet, it is a challenge to estimate indoor exposure ranges or get reliable, consistent reports of individuals’ nocturnal behavior.

Bias may have been introduced by the removal of cases and referents lacking residential addresses that could be matched with geospatial coordinates. Geocoding match rates are far lower for rural areas than for urban areas [42-46]. In general, rural addresses tend to be less specific. Rural delivery routes and post office boxes are often used instead of street addresses, there is more frequent use of unofficial or colloquial place names in rural areas, and roadway reference data for rural areas are less accurate than they are for urban areas [47]. In general, rural-dwelling individuals have lower LAN exposure values, and are more likely to be white (82% as opposed to 66% in urban locations) [19]. Although the unmatched rural locations omitted from analysis may have introduced bias since geospatial location is related to our exposure of interest, the use of only address-level matches ensured an increased accuracy of our participant’s spatial location.

When designing this study, there was no epidemiologic evidence suggesting that exposure to light at night influences lung cancer development. An elevated incidence of lung cancer is not expected with elevated levels of LAN because it is not a hormone dependent form of cancer. Two previous studies investigated the link between levels of LAN and cancer incidence, but no association with lung cancer was reported [9,12]. Recently, Parent et al. (2012) reported novel associations between males ever working at night and cancer risk at several sites, including lung (OR=1.76, 95% CI [1.25, 2.47]) [48]. Although possibly related to LAN, night work could also be associated with exposures to lung carcinogens. If circadian disruption does influence the development of lung cancer, the use of the lung cancer for referents may have attenuated our results. However, little evidence has accrued regarding circadian disruption and lung cancer.

## Conclusions

Our analysis chose to focus on LAN as a potential risk factor for BC, and although results were suggestive of such an association, causation cannot be established. We were unable to measure residential stability, and had no information about residence prior to diagnosis. Due to the latency period of BC, this could potentially impact our results. Furthermore, we had no detailed information pertaining to estrogen receptor status, genetic mutation, and lifestyle factors including obesity, physical activity, alcohol consumption or reproductive history of cases. We attempted to control for parity through the use of county-level birth rates per 1,000 women aged 15–50. More detailed covariate data including residential stability and individual-level reproductive history would further strengthen our study design.

Researchers are yet to fully understand disparities in BC risk, however our analyses provide a plausible environmental explanation for the disparities seen in Georgia. Perhaps mechanisms initiated by LAN exposure may exacerbate pathways by which disease virulence and recurrence are linked. Future work should target the influence of individual-level characteristics (e.g., age, gender, diurnal preference, race, adaptability) and factors related to urbanicity on BC outcomes. Also, it is likely that more accurate proxies of individual artificial light at night exposure will become available as technology advances. The combined use of more refined exposure metrics and more detailed individual-level characteristics should be taken together to explore potential mechanisms behind racial disparities in LAN exposure and cancer outcomes. Similar findings in other geographic regions and populations would further validate our study findings.

## Methods

### Cancer data

Cancer incidence data were obtained from the Georgia Comprehensive Cancer Registry (GCCR) for 2000 to 2007. The GCCR is a statewide population-based cancer registry that collects information on all cancer cases diagnosed among Georgia residents. The GCCR is a participant in the National Program for Cancer Registries and the North American Association of Central Cancer Registries. The GCCR meets national standards for cancer registration and is gold certified with high ratings for data quality and representativeness. For this investigation, the GCCR data were particularly valuable because individual-level location data were available for each subject. A latitude and longitude coordinate for residence location at the time of cancer diagnosis (address-matching) was identified for each cancer diagnosis. The coordinate location assigned to each case was of varying accuracy. Location Codes for Address-Matching (LCODES) are 3–4 text codes that indicated the accuracy level of address-matching. There were 29 LCODES output by the address-matching software, as provided by the GCCR. The first 1–2 characters of the LCODE determine the general level of accuracy and include address-, census block group-, census tract-, and county-level of accuracy. Only cases registering address-level accuracy (AI0-AX3) were included for analysis to minimize misclassification bias. Incidence data were selected for breast (C500:509) and lung cancer (C340:349) sites using the SEER Site Groups Primary Site variable based on International Classification of Diseases for Oncology, 3rd edition (ICD-O-3) coding [49]. Analyses were restricted to female breast and lung cancer cases. Only malignant tumors were included.

### Exposure data

Georgia is positioned in both the northern and western hemispheres in the southeast region of the United States. Georgia averages 110 sunny days a year and the major metropolitan cities include Atlanta, Augusta, Macon, Savannah, and Columbus [50]. Topography begins at sea level and climbs to nearly 5,000 feet above sea level [51]. Data on nighttime stable lights were obtained from the Unites States Defense Meteorological Satellite Program (DMSP) (Image and data processing by NOAA's National Geophysical Data Center, DMSP data collected by US Air Force Weather Agency). Version 4 Defense Meteorological Satellite Program-Operational Linescan System (DMSP-OLS) Nighttime Lights Time Series archive data set consists of high-resolution regional imagery which collects broadband visible-near infrared image data with a nominal spatial resolution of 2.7 km [31]. When collecting nighttime data, telescope pixel values are replaced by Photo Multiplier Tube (PMT) values which are sensitive to radiation from 0.47 - 0.95 um at 10-5 - 10-9 Watts per cm^2^ per sterradian [52].

The satellite imagery for 1992–2007, used in our analysis, was constructed by the DMSP by averaging daily readings of the satellite sensors and removing cloud cover. Nighttime DMSP-OLS images make use of a time series of images to distinguish stable lights produced by cities, towns, and industrial facilities from ephemeral lights caused by fires and lightning [31]. Time series data is composited by using a 1-km grid (finer spatial resolution than that of the input imagery), providing a uniform grid cell size at all latitudes and contiguous land surfaces [31].

A geographic information system (GIS), ArcGIS (ArcMAP software, version 10.0; ESRI, Redlands, Calif) was used to map breast and lung cancer cases by spatial location. ArcGIS technology was used to match individual-level cancer case locations with the LAN levels obtained from satellite images. The task was performed using the spatial analyst tool, “extract values to points,” which joins attributes from one layer to another based on the relative location of features. The “extract values to points” between two data sources was performed as follows: First, a Georgia satellite image of nighttime illumination was imported to the ArcGIS software. The nighttime illumination was characterized by various LAN intensities and displayed by raster units (with a minimum LAN value of 0 (no illumination) and the maximum value of 63 watts per cm^2^ per sterradian (maximum illumination)). Each 1-km raster pixel represented the corresponding ground-level LAN intensity in that geographical region. Three exposure categories were defined for each LAN level in ArcGIS using Jenk’s Natural Break method: low (0–20 Watts per cm^2^ per sterradian), medium (21–41 Watts per cm^2^ per sterradian), and high (>41 Watts per cm^2^ per sterradian). Jenk’s Natural Break method is a data classification method designed to reduce the variance within classes and maximize the variance between classes [53,54]. This method was selected to determine the best arrangement of LAN values into different classes, and create “break points” which minimize each class’s average deviation from the class mean, while maximizing each class’s deviation from the means of the other LAN groups [53,54]. Jenk’s Natural Break method was also used by Kloog et al. in a similar study conducted in 2008. Joined with cases and referents based on their latitude/longitude of residence at cancer diagnosis, LAN levels were extracted for each year of exposure prior to diagnosis using ArcGIS. LAN values were averaged beginning with 1992, the earliest year of exposure data, up until the year of diagnosis. Years prior to diagnosis ranged from 9–16 years, depending on year of diagnosis, 2000–2007. For example, an individual that was diagnosed in 2000 had the previous 9 years of exposure values averaged, 1992 to 2000.

### Covariate data

Individual-level covariates were obtained from the GCCR for 2000 to 2007 and included race, tumor grade and stage, year of diagnosis, and age at cancer diagnosis. County-level (N = 159) covariates were obtained from the American FactFinder data portal, which is an online table generator for U.S. Census data [55]. Prior to model selection via backwards elimination, the full statistical model contained the following county-level variables; average family size, median household income, Metropolitan Statistical Area (MSA) status, average annual particulate matter (PM) 2.5 concentration, births per 1,000 women aged 15–50 in the last year, percent of MSA population living in different residence in 1995, percent of population below the poverty line, percent of African American females, percent of population that are high school graduates or higher, percent of population over 16 in the labor force, and percent of total population in different residence in 1995. Prevalence of cigarette smoking among Georgia women was measured at the Public Health District level (N = 18), from 2000–2004 and obtained from the Behavioral Risk Factor Surveillance System [56]. County metropolitan statistical area classification was obtained from the U.S. Bureau of the Census 2003 Metropolitan Statistical Area (MSA) standards.

### Model building and variable definitions

Manual hierarchical backwards elimination was used to determine the optimal set of covariates. Beginning with the full model, potential confounders were retained in the final model if their removal caused the LAN exposure coefficients’ estimates to change by more than 10%. Based on this procedure, the final model included race, tumor grade and stage, year of diagnosis, age at cancer diagnosis, Metropolitan Statistical Area (MSA) status, births per 1,000 women aged 15–50, MSA population mobility, population over 16 in the labor force, and prevalence of cigarette smoking.

Continuous variables were tested for normality using the Anderson-Darling test for normality and inspection of data distributions and variable plots. Variables that were not normal based on this assessment were categorized for the remainder of the analysis. Categories were either defined as quartiles (tumor stage, grade, births per 1,000 women aged 15–50 in the last year and smoking) or were specified based on knowledge of the variable itself. Individual-level year of cancer diagnosis ranged from 2000–2007. Tumor stage was categorized as local, regional, distant and unstaged. Tumor grade was categorized as well-differentiated, moderately differentiated, poorly differentiated, and other. Racial subgroups were dichotomized as black and white. The sample size within other minority subgroups was too small to support reliable model estimation. MSA status was classified at MSA or non-MSA according to U.S. Bureau of the Census 2003 Metropolitan Statistical Area standards. Among study participants living in Metropolitan Statistical Area or Primary Metropolitan Statistical Area, the percentage living in different residence in 1995 was dichotomized as 0% change or > 0% change. Births per 1,000 women, aged 15–50 years were categorized as <40, ≥40-54, >54-66, and >66. Percent female smokers were categorized as <19.5%, ≥19.5%-21.4%, >21.4%-22.3%, and >22.3%.

### Statistical analyses

Statistical Analysis Software, SAS Version 9.3 (SAS Institute, Cary, NC) was used for data analysis and the probability p < 0.05 was set as the accepted level of statistical significance. The distributions or frequencies of all variables were compared by case status (breast versus lung cancer cases) and Chi-square tests were used to assess statistical differences between groups. Univariable analyses were performed for each individual- and county-level covariate. Final logistic regression models were adjusted for variables on the individual, county, and public health district level, as defined above.

Final unconditional logistic regression analyses were performed with case status as the dichotomous outcome (BC case versus lung cancer referents). Analyses were performed for both races combined and stratified by racial subgroup (whites versus blacks). Measures of association were calculated as odds ratios (OR) with 95% confidence intervals (CI).

## Abbreviations

LAN: Light at Night; BC: Breast Cancer; MLT: Melatonin; IARC: International Agency for Research on Cancer; GA: Georgia; SCN: Suprachiasmatic nuclei; MSA: Metropolitan Statistical Area; OR: Odds ratio; CI: Confidence Interval; GCCR: Georgia Comprehensive Cancer Registry; LCODES: Location Codes for Address-Matching; ICD-O-3: International Classification of Diseases for Oncology, 3rd edition; DMSP-OLS: Defense Meteorological Satellite Program-Operational Linescan System; PMT: Photo Multiplier Tube; GIS: Geographic information system; GCCR: Georgia Comprehensive Cancer Registry; AA: African American.

## Competing interests

The authors declare that they have no competing interests.

## Authors’ contributions

SEB conceived of the study, participated in its design, produced ArcGIS maps, assisted with statistical analyses and drafted the manuscript. SEW carried out statistical analyses, participated in the study design, produced ArcGIS maps, and drafted the manuscript. JB helped to draft the manuscript. RB provided the cancer data. JEV participated in the design of the study, drafted the manuscript and coordinated the project. All authors read and approved the final manuscript.

## Authors’ information

SEB is pursuing her doctorate in Health Services Research, Management and Policy at the University of Florida. SEW is an Assistant Professor in the Department of Epidemiology and Biostatistics, College of Public Health, University of Georgia. JB is an Associate Professor in the Department of Epidemiology and Biostatistics, Cancer Prevention & Control Program, University of South Carolina. RB is the Director of the Georgia Comprehensive Cancer Registry. JEV is Head of the Department of Epidemiology and Biostatistics, College of Public Health, University of Georgia and a University of Georgia Foundation Professor.
